# Gastric perforation leading to the diagnosis of classic Ehlers–Danlos syndrome: a case report

**DOI:** 10.1186/s13256-021-03108-6

**Published:** 2021-10-26

**Authors:** Ahad E. Alotaibi, Ohood H. AlAamer, Mohammed A. Bawazeer, Ali A. Alzahrani

**Affiliations:** 1 College of Medicine, Princess Nourah Bint Abdulrahman University, Riyadh, Saudi Arabia; 2 Department of General surgery, King Saud bin Abdulaziz University for Health Sciences, King Abdullah International Medical Research Center Ministry of National Guard Health Affairs, Riyadh, Saudi Arabia; 3 Department of Critical Care Medicine, King Faisal Specialist Hospital and Research Centre, Riyadh, Saudi Arabia

**Keywords:** Ehlers–Danlos syndrome (EDS), Gastrointestinal perforation, Volvulus, Marfan’s syndrome

## Abstract

**Background:**

Ehlers–Danlos syndrome is a clinically and genetically heterogeneous group of heritable connective tissue disorders caused by a defect in collagen synthesis and structure. The vascular subtype (Ehlers–Danlos syndrome IV) is reported to be associated with a higher incidence of gastrointestinal perforations. The most reported site of perforation is the colon, followed by the small bowel. Perforation of the stomach is very rare, and there are no reported cases to date of classic types I and II.

**Case presentation:**

We present the case of a 14-year-old Saudi girl who visited our emergency department with abdominal pain and vomiting. Initially, she was diagnosed with gastroenteritis and discharged once her condition stabilized. After 48 hours, she developed severe abdominal pain with recurrent vomiting and peritonitis evident on clinical examination. Initial abdominal x-ray failed to show any free air; however, enhanced computed tomography revealed free air and contrast extravasation in the proximal gut. During exploratory laparotomy, a large perforation was found on the anterior wall of the stomach due to the underlying ischemia. The posterior wall had ischemic mucosa with an intact healthy serosa. A free-hand partial gastrectomy was performed to resect all ischemic parts of the stomach. Detailed examinations and laboratory workup were carried out after the surgery to figure out the possible underlying cause. The clinical findings during the physical examination supported marfanoid features. Marfan’s syndrome and related disorders sequencing panel was requested, and Deoxyribonucleic acid (DNA) samples were sent. Given results were supporting the diagnosis of classical Ehlers–Danlos syndrome, the patient was labeled as a case of Ehlers–Danlos syndrome. During the postoperative period, she developed a wound infection that was managed successfully with vacuum-assisted closure dressing. She recovered well without gastrointestinal sequelae in the 4 years of follow-up.

**Conclusions:**

Heritable systemic connective tissue diseases must be given serious consideration in young patients with unusual spontaneous perforation. Such patients might develop life-threatening conditions that require immediate intervention. Hence, correct and timely diagnosis is important to prepare for the anticipated complications.

## Background

Ehlers–Danlos syndrome (EDS) comprises a wide spectrum of overlapping hereditary disorders of the connective tissues. EDS results from defects in the synthesis of collagen, which might lead to a wide range of clinical presentations affecting the skin, ligaments, joints, blood vessels, and internal organs with variable extent [1–3]. EDS is clinically classified according to the 1997 Villefranche nosology into three major groups: classical, vascular, and hypermobility. Other types include kyphoscoliosis, dermatosparaxis, and arthrochalasia, which are extremely rare [3, 4]. Genetically, most cases of classical EDS are caused by mutations in one of the two genes encoding collagen type V (*COL5A1*, *COL5A2*). Vascular EDS is caused largely by mutations in *COL3A1*, encoding collagen type III.

The genetic changes causing the hypermobility-type EDS remain largely unknown, and are predicted to be heterogeneous [3, 5]. Since the EDS types have phenotypic heterogeneity and clinical overlap, clinical evaluation alone is often not definitive. Despite genetic testing, the majority of EDS cases do not have a molecular diagnosis. Thus, some patients might exhibit a delayed presentation or might present with complications [3, 4].

The clinical presentation in patients with EDS varies depending on the subtype. Gastrointestinal (GI) symptoms are observed in all types of EDS. The most commonly reported symptoms are abdominal pain, nausea, and constipation with a wide range of severity among the subtypes of EDS [6–9]. A systematic review by El Masri *et al*. comprising 31 patients (27 case reports, four retrospective studies) showed that life-threatening digestive complications are usually seen in the vascular type of EDS (EDS IV). Spontaneous GI perforations are the most common complications. The colon is the predominant site of perforations, particularly the sigmoid colon, as it contains a high volume of collagen. This is followed by perforation of the small bowel and the upper rectum. There are limited case reports about esophageal rupture and to a lesser extent about gastric perforation [10]. Gastric symptoms in patients with EDS range from recurrent epigastric discomfort to severe bleeding [10]. Peptic ulcers and their complications have been described mainly in patients with vascular-type EDS [10]. Gastric perforations are rarely documented and are mostly related to trauma [10].

In this report, we present an uncommon case of spontaneous gastric perforation with undiagnosed classical EDS and marfanoid features. Written informed consent was obtained from the patient and her father to publish this report and the accompanying images.

## Case presentation

**Fig. 1:**
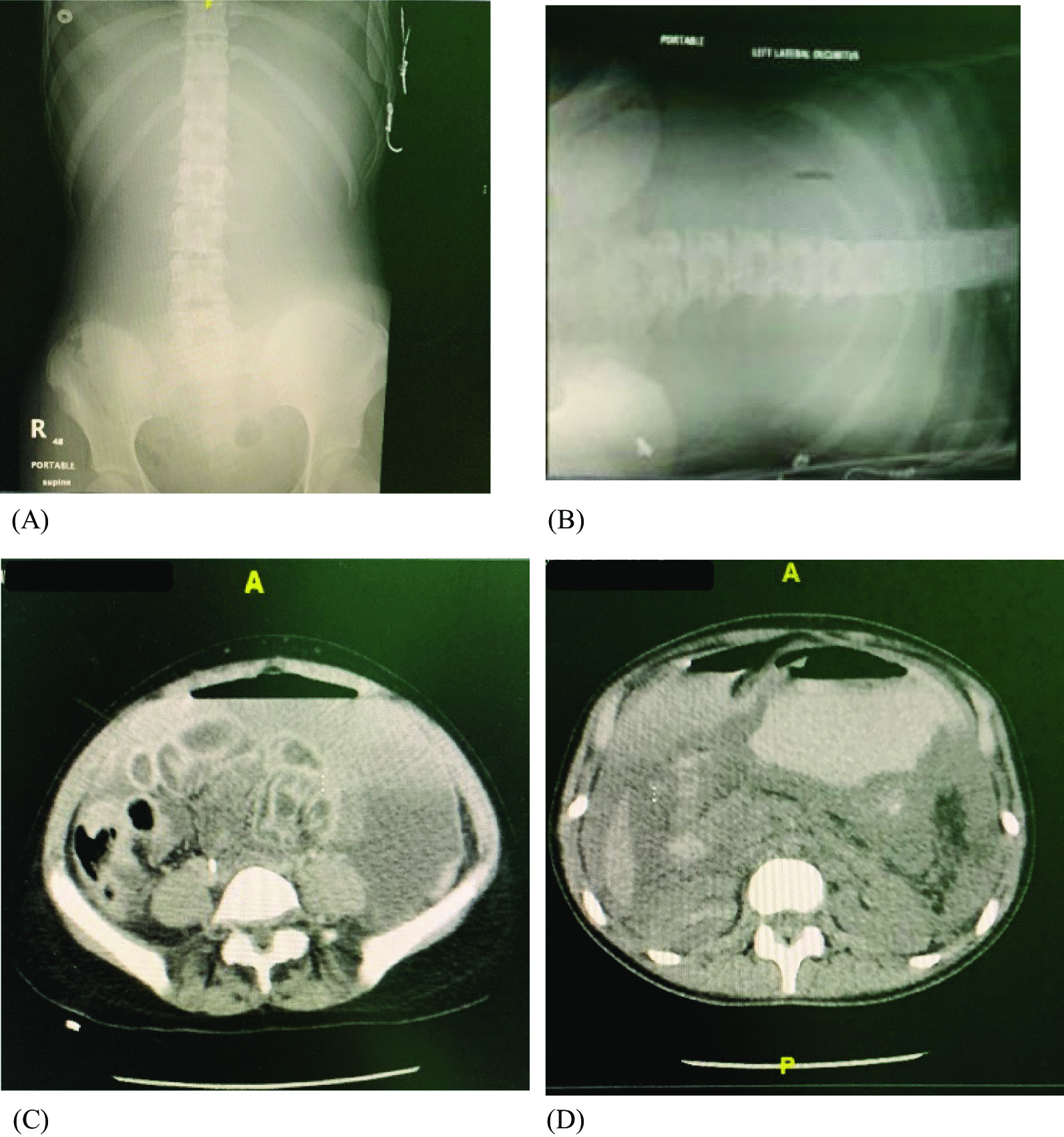
**A** Abdominal x-ray supine position; **B** lateral decubitus. **C**, **D** Computed tomography image of the abdomen and pelvis with contrast with findings of contrast extravasation

We present the case of a 14-year-old Arab female patient who presented to our emergency department at 2:45 a.m. with epigastric abdominal pain and vomiting twice for 3 hours. She was previously healthy and was not known to have any medical or surgical conditions. Her family history was also negative for any congenital diseases. The patient had stable vital signs, with a temperature of 37 °C, heart rate of 91 beats per minute, blood pressure of 126/72 mmHg, respiratory rate of 20 breaths per minute, and oxygen saturation of 99% on room air. The abdomen was soft and nontender. She was initially managed as a case of gastroenteritis with symptomatic improvement and was sent home when her condition improved. She presented again in the morning of the third day after the initial presentation with worsening abdominal pain and repeated episodes of vomiting. On clinical examination, her blood pressure was 100/58 mmHg with tachycardia up to 130 beats per minute; however, she was afebrile, and her temperature was 36.2 °C. Abdominal examination revealed diffuse tenderness on superficial and deep palpation, rebound tenderness, and guarding consistent with generalized peritonitis. Radiological images included abdominal x-ray in the supine and lateral decubitus position (Fig. 1A, B). Initial resuscitation with intravenous fluids showed a good response. After stabilization, she was transferred to computed tomography (CT) for further investigation of the underlying pathology (Fig. 1C, D). Laboratory tests on admission were as follows: white blood cells 24.8 × 10^9^/L; hemoglobin 16.5 g/dL; platelets 1008 × 10^9^/L; red blood cells 6.33 × 10^12^/L; international normalized ratio (INR) 1.28; prothrombin time 14 seconds; lactic acid 6.80 mmol/L; amylase 1015 U/mL; total bilirubin 20.7 µmol/L; direct bilirubin 8.7 µmol/L; creatinine 227 μmol/L; blood urea nitrogen (BUN) 12.5 mmol/L; and anion gap (AGAP) 25 mEq/L. However, the patient’s condition deteriorated on returning from the CT table. Her vital signs were unstable, with a temperature of 37.5 °C, respiratory rate of 30 breaths per minute, heart rate more than 130 beats per minute, and blood pressure less than 100/50 mmHg. Hence, she was taken to the operating room (OR) for an emergency laparotomy.

**Fig. 2:**
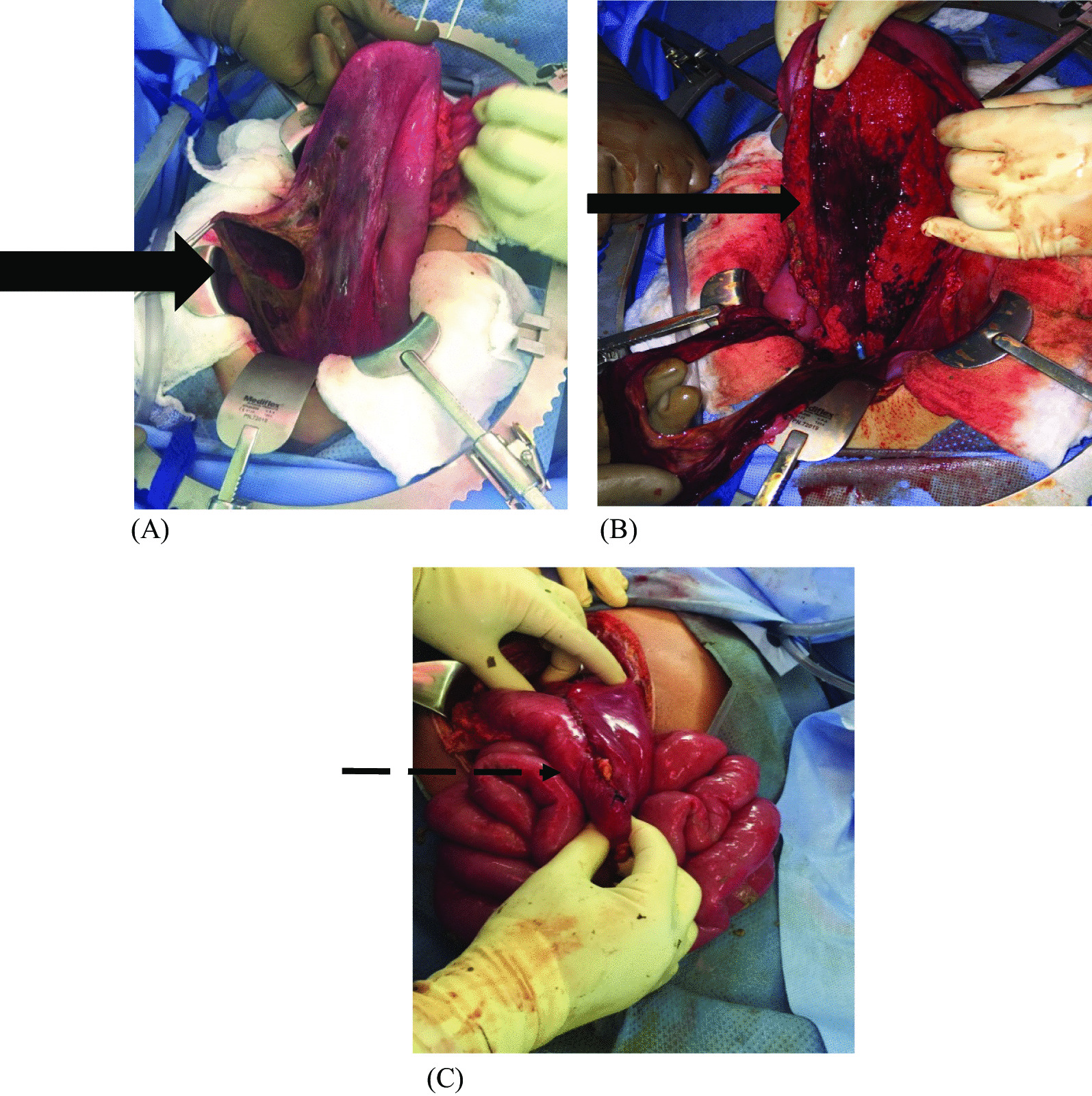
**A**, **B** Intraoperative laparotomy showing completely ischemic anterior and posterior wall of the stomach with gastric perforation, 2 × 2 cm hole within the ischemic wall in **A** (bold arrow) and internal gastric wall in **B** (thin arrow). **C** Partial gastrectomy with gastrojejunostomy anastomosis (dotted arrow)

On exploratory laparotomy, a large (2 × 2 cm) perforation was found in the anterior wall of the stomach with underlying ischemia, as shown in Fig. 2. In addition, ischemic changes were noted over the mucosa of the posterior wall, although the serosa appeared normal. Free-hand partial gastrectomy was performed. The necrotic edges of the anterior wall were excised, and the remnant healthy bleeding edges were sutured together (Fig. 2). A Jackson–Pratt drain was placed, and the abdomen was closed temporarily with a planned second-look laparotomy combined with intraoperative endoscopy to evaluate the posterior wall vascularity. The patient was then transferred to the intensive care unit (ICU) intubated, with stable hemodynamics and off vasopressors.

**Fig. 3:**
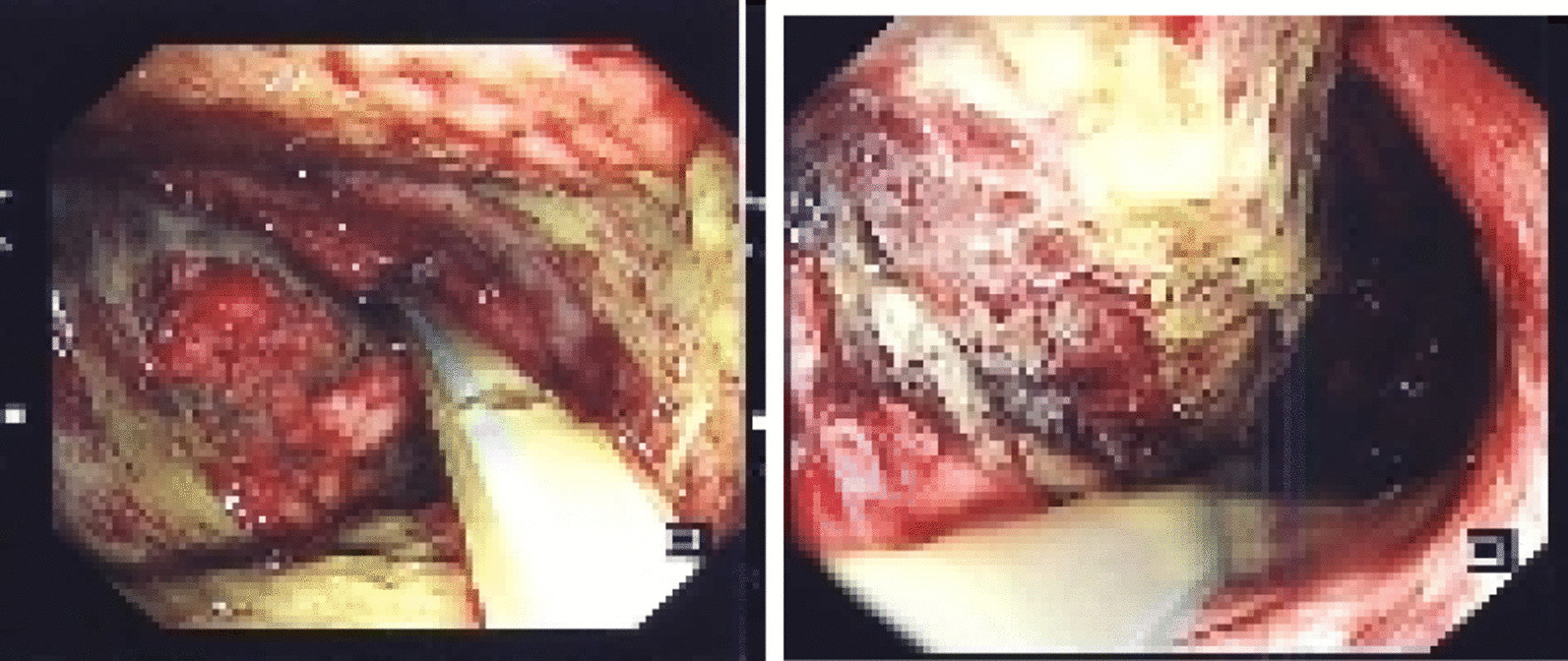
Intraoperative endoscopy, with findings of necrotic mucosa

After 48 hours, she was taken back to the operating room for a second-look laparotomy with intraoperative endoscopy. Endoscopy findings were consistent with limited mucosal ischemic changes (Fig. 3). Moreover, the stomach serosa was healthy with no further ischemic changes and an intact suture line. A jejunostomy feeding tube was inserted, followed by definite abdominal wall closure. Postoperatively, the patient remained in the ICU for monitoring and was successfully extubated. Jejunostomy tube feeding was initiated, and the patient was transferred to the regular surgical floor. Upon initiation of oral diet, the patient gradually started consuming six small meals per day owing to the reduced size of the stomach.

**Fig. 4:**
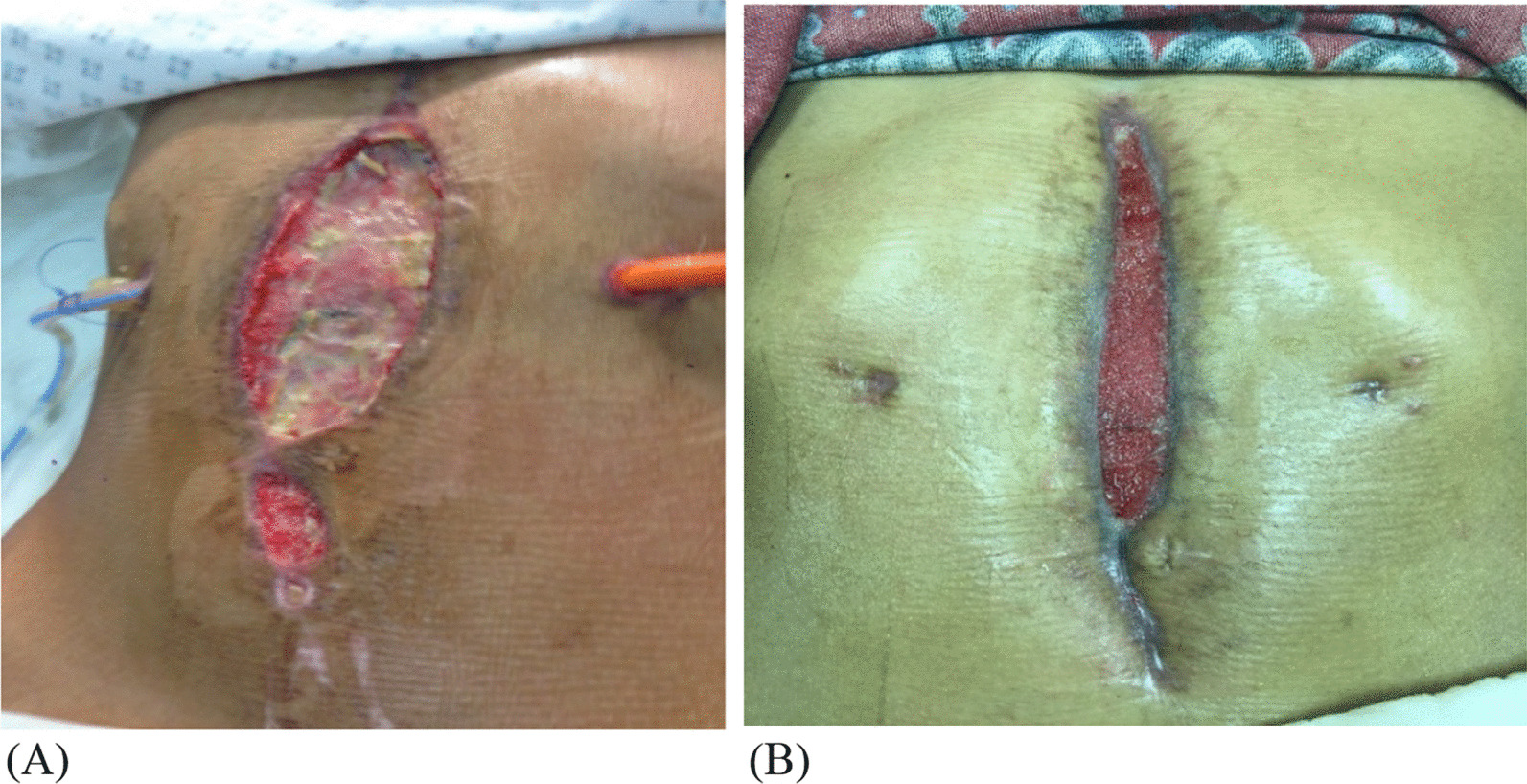
**A** Jejunostomy feeding tube with superficial surgical site infection. **B** Interval removal of the feeding tube with wound progression vacuum-assisted closure (VAC)

The postoperative course was complicated with superficial surgical site infection and improper wound healing, which was managed with antibiotics, and the wound was managed using a vacuum-assisted closure (VAC) device. Figure 4 shows the wound progression while on VAC dressing. Pathology report was done on two specimens: gangrenous part of ischemic stomach (black, dusky soft tissue, measuring 2 × 1.3 × 0.3 cm with red-tan cut surface), and part of stomach with unhealthy mucosa (gray-tan hemorrhagic, soft tissue measuring 2 × 1.3 × 0.3 cm with red-tan cut surface). The preoperative diagnosis was perforated viscous, whereas the postoperative diagnosis was ischemic anterior wall of stomach and perforation with no obvious etiology.

This presentation is concerning due to the underlying systemic conditions. A detailed clinical examination was performed postoperatively. The examination supported marfanoid features, including long fingers and positive wrist and thumb signs, with mildly hyperextensible skin. Therefore, Marfan’s syndrome and related disorders sequencing panel was requested, and DNA samples were obtained. The result detected a heterogeneous variant of uncertain clinical significance in exon 6 of the COL5A1 gene, c.805G>A (p.Glu269Lys), which supports the diagnosis of classical EDS. In addition, rheumatological workups were performed, and all were within the normal range.

The patient remained in the hospital for a couple of weeks postoperatively for wound care management and nutritional support and was discharged after removal of the Jackson–Pratt (JP) drain and jejunostomy feeding tube. On follow-up, the patient was doing well from a nutritional and surgical perspective as well as in her school performance.

## Discussion and conclusions

As the diagnosis and management of EDS is challenging, prompt attention to anticipate the possible complications and avoid further damage is necessary [11]. Patients with EDS have a wide range of GI symptoms. Frequently reported symptoms include nausea, vomiting, heartburn, constipation, abdominal pain, and inflammatory bowel syndrome, varying in severity between the different subtypes [6, 8]. However, GI complications are more common in patients with the vascular form of EDS, and they are at a high risk of spontaneous perforation [10–12]. Other major forms of type I, II, and III develop recurrent hernia as the most common complication, which can be related to skin hyperextensibility [6, 10, 11].

Spontaneous bowel perforation is a well-documented and potentially life-threatening complication of EDS, especially in the vascular type, with more than 200 reported cases in the English literature. The commonest reported site of perforation is colonic perforation, specifically sigmoid colon, followed by small bowel perforation, and it is the commonest presentation in vascular-type EDS [6, 10, 13–19]. Furthermore, stomach perforation is not common and is mostly related to traumatic injury [10, 20, 21]. One of the important factors leading to gastric perforation despite the rich blood supply, and to a lesser extent compared with the vascular type IV, could be secondary to marfanoid habitus that can lead to gastric volvulus due to the abnormality of gastric ligaments and diaphragmatic hernia, leading to a higher probability of compromised blood supply and subsequent wall defects and perforation [21–23]. The rarity of such presentations presents a unique challenge in management.

As EDS patients with perforation of a hollow viscus usually present in an acute state, immediate surgical intervention is often required. Surgical management of patients with vascular EDS who develop acute GI complications such as bleeding or perforation has been described in the literature. These approaches range from conservative nonsurgical management of intestinal perforation to more conventional surgical management with resection of the affected segments of the gut [19, 24–26]. Some patients have undergone segmental bowel resection and primary repair with stoma creation followed by later attempts at reversal [17, 27].

Finally, a diagnosis of this hereditary condition has implications for the patient’s family, and genetic testing needs to be offered to the living relatives. As the patient has a 50% probability of having an affected child, reproductive counseling as well as predictive, diagnostic, and prenatal testing should be made available.

## Conclusion

GI involvement in EDS and Marfan’s syndrome is common, ranging from benign to life-threatening conditions. It is crucial to prevent fatal complications and to identify the subtype of EDS to predict morbidity and mortality. In any young patient presenting with bowel perforation anywhere in the GI tract or any vascular accident without a known etiology, EDS needs to be considered on the differential diagnosis list after excluding other causes. Although EDS is a rare etiology, more and more cases of EDS are being reported to be a cause of bowel perforation. The patient must be educated about the involvement of all body organs and the possible complications. In addition, family counseling and genetic tests are obligatory in these cases.

## Data Availability

Not applicable.

## Abbreviations

CT: Computed tomography
ED: Emergency department
EDS: Ehlers–Danlos syndrome
GI: Gastrointestinal
ICU: Intensive care unit
VAC: Vacuum-assisted closure
